# Psychosocial aspects of preconception consultation in primary care: lessons from our experience in clinical genetics

**DOI:** 10.1007/s12687-012-0095-z

**Published:** 2012-05-15

**Authors:** S. Riedijk, G. Oudesluijs, A. Tibben

**Affiliations:** 1 Department of Clinical Genetics, Erasmus Medical Centre, P.O. Box 2040, 3000 AC Rotterdam, The Netherlands; 2 Department of Clinical Genetics, Leiden University Medical Centre, Leiden, The Netherlands

**Keywords:** Preconception consultation, Psychosocial aspects

## Abstract

To date, little is known about the psychosocial aspects of preconception consultation (PCC) in primary care. PCC in primary care is appropriate for couples and individuals with a reproductive wish. In PCC, non-genetic and genetic risk factors may be identified. Focusing on non-genetic and genetic risk factors in PCC requires the use of different counselling strategies and tools in optimizing the outcome of pregnancy. Addressing lifestyle alterations requires directive counselling, whereas addressing increased genetic risk and its subsequent reproductive options requires non-directiveness. When an increased genetic risk is detected, couples should be informed about their possibilities for not passing on a disease allele. Depending upon the various modes of inheritance and reproductive options, couples may face a variety of psychosocial challenges. This paper aims to provide insights into the psychosocial impact of the genetic aspects of PCC by drawing upon literature and clinical experience in the Clinical Genetics department. Furthermore, this paper provides consideration for future developments regarding preconception genetic screening.

## Preconception care

Preconception care is one of the main instruments of high-income countries to reduce stillbirth rates (Flenady et al. 2011). In 2007, the Dutch Health Council recommended to initiate preconception care by means of a central programme. Since 2006, a rapidly growing number of midwifery practices have started offering preconception consultation (PCC) in the Netherlands. Preconception care has thus become more integrated in primary health care, thereby increasing the uptake. The sole indication for preconception care is the wish or consideration to become pregnant. PCC may focus on lifestyle and work and living environment issues, medicine use and advice to use folic acid supplements, advanced parental age, consanguinity, smoking/alcohol/drugs (ab)use, teratogens, infectious diseases, chronic disease of the woman, previous gynaecological problems (miscarriages, labour problems), congenital anomalies or hereditary disease of the woman or man, a previous child with a congenital anomaly or hereditary disease, family history with a congenital anomaly or a (possible) hereditary disease (Atrash et al. 2008). Thirty couples receiving PCC in a Dutch trial had an average of six risk factors per couple (De Jong-Potjer et al. 2003). According to a 2010 WHO report on community genetics in middle- and low-income countries, genetic components of primary preconception care include: detection of genetic risks through family history, addressing the issue of consanguinity if relevant, explaining programmes of prevention of congenital disorders and genetic diseases that exist in the community, and genetic counselling as appropriate (Al-Arrayed et al. 2010). In the Netherlands, the request has been made to add preconception carrier screening of cystic fibrosis (CF) and heamoglobinopathies (HbPs) to primary preconception care (Cornel et al. 2011; van Elderen et al. 2010). In 2007, the advisory report ‘Preconception care: a good beginning’ advised that preconception screening may be offered for CF and HbPs in the Netherlands (Netherlands HCot 2007). To date, this screening has not been implemented.

If preconception genetic screening for autosomal recessive disorders such as CF and HbPs is offered in the setting of PCC, then couples should receive adequate counselling. Couples should be informed about what carriership implies for them personally, for their families and for their reproductive options. Depending upon the chosen reproductive option, couples may face a variety of psychological challenges. To date, research focusing on the psychosocial impact of genetic counselling in preconception care is scarce. This paper aims to provide insights into the psychosocial impact of genetic counselling in preconception care by drawing upon literature and clinical experience in the Clinical Genetics department. This paper will focus on two themes regarding genetic counselling in preconception care: counselling and its psychological impact.

## Counselling of non-genetic and genetic aspects in PCC

When non-genetic risk factors are identified in PCC, information is provided to enable couples to change their behaviour in ways that are beneficial to the pregnancy. Pregnancy may be positively influenced by starting a healthy diet, losing weight, taking folic acid supplements, tobacco, alcohol and drugs cessation, and taking part in regular exercise. As the stages of change model illustrates (Prochaska et al. 1994), in order to adjust behaviour, more than information is required. A counselling aimed at changing behaviour should be directive and should comprise an assessment of the stage of change a person is in, and following the stage either information, or more practical advice, empowerment or reinforcement is necessary.

When an increased genetic risk is identified in PCC, information is provided to enable couples to make informed decisions about their options for not passing on a disease allele to their offspring or to reduce the risk of an affected live born child. Contemplating the impact of (possibly) developing a (un)treatable disease in the future, and considering the options for not passing on a disease allele require that the counselee is offered information and a non-directive discussion in which the counselee works through the various scenarios she or he might be facing. She or he then needs to evaluate which of the options fits best with her or his capacity and personal values. An indication for referral to a clinical genetics centre may be identified during PCC. A couple will then have to decide whether or not they wish to engage in further genetic counselling. Given the possible consequences of risk estimation or genetic testing, it is important that a couple is offered non-directive counselling as part of genetic PCC to assist them in this decision as well.

Thus, focusing on genetic and non-genetic risk factors in preconception counselling requires the use of different counselling strategies, namely directive and non-directive counselling, respectively, and different interventions in optimizing the outcome of pregnancy. This is important because it implies that the counsellor in PCC will have to be able to switch counselling strategies as appropriate during the consultation.

## Reproductive options

If couples are at increased genetic risk or are offered genetic preconception screening, they should be informed about the reproductive options that are available to them. When couples are proven carriers of a disease allele, they may opt for prenatal diagnosis (PND), preimplantation genetic testing (PGD), sperm or egg cell donation, natural conception or refraining from having children. The non-directive approach in the counselling implies that the counsellor does not have a preference with regard to engaging in genetic screening or with respect to reproductive options. The counsellor aids the couple in discovering what the best option is for them.

## Prenatal diagnosis

In PND, chorionic villus sampling and amniocentesis are invasive methods to collect foetal material (Raymond et al. 2010). Both methods carry a small risk of miscarriage. Chorionic villus sampling is possible at 10–13 weeks gestation, and the test result may be known before 14 weeks gestation. This implies that pregnancy may be ended by means of curettage. Amniocentesis is performed around 15–17 weeks gestation, and the test result may be known after approximately 2–3 weeks. In case of an affected foetus, the pregnancy may be ended by inducing labour in a hospital setting.

When there is an increased risk for a structural congenital anomaly in offspring, PND by advanced ultrasound examination is frequently possible. Detecting an anomaly provides the opportunity to influence the course of the pregnancy. However, normal ultrasound findings are not informative for all anomalies/disorders (e.g. anal atresia or learning disabilities). Our clinical impression is that this subgroup of at-risk individuals may experience a significant amount of distress because they know there is an increased risk of affected offspring, but they have no options to reduce this risk. In the Netherlands, couples may decide to end the pregnancy by labour induction before 24 weeks of gestation.

In the near future, it is expected that non-invasive prenatal diagnosis (genetic analysis on foetal DNA extracted from maternal blood) will be possible for a limited number of indications. The major advantage of this type of PND is the avoidance to a large extent of the abortion risk.

Research showed that the psychological impact of pregnancy termination increased as gestational age advanced (Davies et al. 2005). Overall, women experienced intense grief, trauma, psychological complaints and pressure on the partner relationship after late pregnancy termination (>16 weeks gestation) and occasionally regret (Korenromp et al. 2006). While most women were able to resolve their grief, more than one third of the women still experienced elevated levels of trauma and grief up to 4 years after the pregnancy termination (Davies et al. 2005; Korenromp et al. 2005a; Hunfeld et al. 1997; Korenromp et al. 2007). Because of the impact of ending a desired pregnancy, it is important that couples are prepared for all the issues involved in the decision whether or not to opt for PND. The severity of the condition, its treatability, the family history of the condition and the couples’ attitude towards pregnancy termination all contribute to the couple’s perception of the disease and their motivation for PND. Couples who have lost relatives or witnessed the symptoms of a disease may be more motivated to prevent passing on the disease allele and opt for PND, and may experience fewer doubts than couples who have not witnessed the disease. Couples do not always agree on whether they wish to have PND. In our clinical experience, men are more inclined to opt for PND than women. Moreover, research has shown that women and men also respond differently to pregnancy termination. Women experienced more grief and trauma from pregnancy termination than men, but women receiving partner support generally coped better (Korenromp et al. 2005b; Geerinck-Vercammen and Kanhai 2003). For the quality of the partner relationship, it is important that couples resolve their differences and decide about PND in unison.

## Preimplantation genetic diagnosis

In our experience, couples generally perceive PGD as an option when ending a pregnancy is not an option or when they already need IVF due to decreased fertility. In the Netherlands, there is a committee reviewing PGD requests. As a guideline, each condition that is an indication for PND is also an indication for preimplantation genetic diagnosis (PGD); however, there are exceptions (Geraedts and De Wert 2009). PGD involves in vitro fertilization, testing the embryo genetically and transferring it to the uterus only if it is not carrying the disease allele (van Rijn et al. 2011). PGD requires considerable time and effort, with a pregnancy rate of around 15–20 % each trial (http://www.pgdnederland.nl/). A recent pilot study indicated that women undergoing PGD had increased anxiety scores around the time of fertilization and pregnancy testing, but did not take into account the whole procedure of PGD (e.g. being on the waiting list whilst experiencing a strong reproductive wish, etc.) (Karatas et al. 2011). In our clinic, couples having experienced PGD indicated they found PGD quite burdensome. Couples are offered psychosocial counselling during the PGD process.

## The psychological function of pregnancy

Surprisingly, few studies have evaluated the psychological impact of preconception counselling. In order to grasp the possible psychological impact of being confronted with genetic risk during preconception consultation, it is important to understand the psychological function of pregnancy. It may be assumed that couples, who wish to be informed about genetic risks, express their wish to have children and at the same time feel responsible for the future child’s health and welfare. Hence, from a psychodynamic point of view, the couple’s decision to plan a pregnancy represents a developmental milestone and a psychosocial crisis (Leon 1992a). First, the outlook on parenthood might give each of the couple an independent sense of adult identity with different perspectives for the prospective mother and father. In case of hereditary risks, we have often observed that the mother is particularly concerned with the welfare of the future child, whereas the father feels protective towards the entire family system (e.g. the well-being of the other children in the family, maintenance of quality of life of the family). Second, to both prospective parents, a pregnancy means an enhancement of the self and one’s own importance, and achievement of omnipotent feelings, which may be challenged when the pregnancy is threatened by hereditary risks. Third, longing for a pregnancy also implies that the couple wishes to create a new object relationship which underlines the increasing identification with parental figures in past and present (Leon 1992b). All of these psychodynamic functions of pregnancy may be threatened when couples discover their genetic risk.

When couples are offered PCC and are informed about the genetic risks for future children, they become aware of the tension between the desire to have, nurture and raise a child on the one hand and their sense of responsibility on the other hand. Parents may experience guilt feelings towards (future) offspring (Strømsvik et al. 2009; van Oostrom et al. 2007; Klitzman et al. 2007).

Confrontation with genetic risks and appeal to the feelings of responsibility towards the future child and others involved may attenuate the desire for a pregnancy. Moreover, the marital relationship may be challenged when one member of the couple feels differently than the other with regard to the need to have PCC and the subsequent management (reproductive screening/testing) options, especially if one member of the couple has multiple risk factors and difficulties to adapt. While PCC has a highly informative character for couples, in general, it can be said that PCC may alter the subsequent pregnancy experience. Once informed of one’s genetic risks, the idealized representation of pregnancy dissipates. The information that a genetic risk exists and the availability of genetic testing or screening may increase the social pressure to seriously consider and apply for screening (van Elderen et al. 2010).

## The psychosocial impact of genetic risk and carriership

Regardless of whether preconception screening for certain autosomal recessive disorders is implemented, couples may be confronted with a genetic risk during PCC based on their family history. Couples who attend the Clinical Genetics department are anticipating learning about their genetic risk, whereas learning about an increased genetic risk during PCC may catch couples by surprise. Studies evaluating the psychological impact of PCC are scarce. The few studies that were conducted expected PCC to elicit anxiety; however, it was found that anxiety levels did not increase after preconception counselling (de Weerd et al. 2001; De Jong-Potjer et al. 2006), and in contrast, some subgroups experienced a decline in anxiety after preconception counselling.

In Clinical Genetics, more research has focused on the psychological impact of genetic risk and carriership. Various modes of inheritance also present with a variety of psychosocial issues that may be relevant in aiding couples deciding about engaging in further genetic testing. Furthermore, depending upon the mode of inheritance, different reproductive options may apply that each have differing psychological challenges. The PCC counsellor should be aware about these issues to adequately prepare couples for the decisions and implications that may follow genetic screening or testing.

In case of a balanced chromosomal rearrangement (e.g. translocation, inversion) in the family, couples may present for carriership testing. These couples may be referred for PCC after recurrent miscarriage or a previous affected child (due to an unbalanced chromosomal rearrangement). Depending on the type of balanced chromosomal rearrangement in the parent, recurrence risk for an unbalanced chromosomal rearrangement in the offspring may be lower or higher (McKinlay Gardner and Sutherland 2004). It is our experience that some couples with recurrent miscarriage and couples with a previous child with a de novo unbalanced chromosomal rearrangement may hesitate about prenatal diagnosis (PND) due to the (small) miscarriage risk of invasive prenatal diagnosis. Some of them express the wish to perform advanced ultrasound examination, which is not the golden standard for chromosomal aberrations. In addition, women with a high recurrence risk of miscarriage may experience high levels of anxiety (Vansenne et al. 2011).

In case of X-linked (recessive) disorders such as Fragile X or Duchenne muscular dystrophy, a woman may be carrying a mutation from which she experiences no or mild symptoms, but that may cause her sons to be severely affected. Although daughters often are healthy, prospective mothers may find it undesirable for their daughters to be carriers. In the clinic, we have observed women who objected to passing on their reproductive issues to their daughters. Mothers who were proven carriers with an affected child were more inclined to change their reproductive plans (Lewis et al. 2011). Research has shown that mothers of children affected by X-linked disorders had a rather strong tendency to experience feelings of guilt and self-blame, often reinforced by the father who may blame the mother as well (James et al. 2006). Given the difficulties carrier women have in disseminating the information to at-risk relatives, recommendations are to offer women support to ensure that relatives with a reproductive wish are informed in a timely manner about their own risk for transmitting the disease allele (van Rijn et al. 1997).

In case of an autosomal recessive disorder in the family, such as cystic fibrosis (CF), couples may present for carriership testing. These couples often are aware of the disease because of their family history. Generally, heterozygosity, in case of CF, has no consequences for the health of the prospective parents (Read and Donnai; in this issue). Studies into screening for CF found that carriers were not greatly distressed about their personal test result. However, if both partners were carrying a CFTR mutation, they may feel distressed about the increased risk for their offspring (Watson et al. 1992). Another study found that carriers reported no impact of the test result on their reproductive plans (Henneman et al. 2002). In case of screening, there is generally no positive family history of CF and couples may have a less vivid image of what CF may be. Studies showed that parents of a child with CF choose to have PND in 20 to 65 % of cases (Evers-Kiebooms et al. 1990; Borgo et al. 1992; Jedlicka-Köhler et al. 1994), but carrier–carrier couples opted for PND in 28 out of 31 cases (90 %) (Super et al. 1994; Brock 1996). Couples may be less prepared to accept a miscarriage risk when they have already had the experience of bearing and raising a child. In case of autosomal recessive disorders, couples may have trouble understanding their reproductive risks (James et al. 2006). Several studies have consistently reported that recall and understanding of genetic risk is poor (Austin 2010; Smerecnik et al. 2009).

When one of the prospective parents is at increased risk of transmitting a known autosomal dominant disorder such as Huntington disease (HD), carrier testing is an option in order to determine whether one’s offspring is at increased risk as well. Genetic counsellors view the discussion of reproductive options as one of the five main themes of the counselling for HD (Hines et al. 2010). These individuals often indicate that in the absence of a reproductive wish they would not opt for testing. However, the test not only generates knowledge about the risks of the child but also about the future health of the prospective parent. In counselling, much attention is paid to the psychosocial aspects of receiving an unfavourable test result for oneself. Positive carrier testing could result in lowered self-esteem, stigmatization, discrimination and denial of health and life insurance, and employment opportunities (Markel 1992; Lakeman et al. 2009).

Couples of whom one is an HD-mutation carrier might decide not to postpone starting a family. However, they may neglect that the children will be exposed to potentially intrusive or even traumatic experiences with an affected parent in early childhood. Research has shown that individuals exposed to an affected parent early in childhood more often had an insecure attachment representation, which is associated with worse adult functioning (Van der Meer et al. 2006). This issue may be addressed in genetic PCC.

Female carriers of the breast cancer 1 or 2 disease allele represent a special case for genetic PCC. These women are at increased risk for breast and ovarian cancer, raising three reproductive issues: the use of contraceptives, preventive surgery and breastfeeding, and the possibility of prenatal diagnosis (Quinn et al. 2009), all of which should be addressed in genetic PCC.

There is strong scientific support for the idea that major psychiatric illnesses such as bipolar disorder, autism, alcoholism, schizoaffective disorder and schizophrenia are caused by the combined influences of both genetic and environmental contributions (Austin and Peay 2006). Both affected and healthy individuals may have concerns about passing on susceptibility for psychiatric conditions to their offspring. The combined influence of genetics and environment may easily lead to misunderstanding of genetics and over- or underestimation of risks. Consequently, this may lead to decisions which would otherwise not be made. If individuals with a psychiatric disorder request genetic PCC, special attention should be paid to the tension between ‘desire for a child’ and responsibility as individuals with a psychiatric disorder may have above average problems with information processing, balancing considerations and emotion regulation.

## Discussion

When couples engage in PCC, they may be confronted with increased genetic risk based on their family history. It is expected that in the near future, PCC will also comprise the offer of carrier screening for CF and HbPs.

PCC providers should be aware of the different counselling strategies that are appropriate when focusing on non-genetic and genetic risk factors in PCC. When focusing on non-genetic risk factors, directive counselling is a more adequate approach influencing behaviour (medicine use, healthy lifestyle, drug cessation, etc.). When focusing on genetic screening and (the consideration of) testing, a non-directive approach is necessary. Non-directiveness enables couples to process relevant information and reach their own decision regarding whether they wish to engage in genetic screening or testing. Even though studies demonstrated that carrier screening for CF and HbPs did not elicit adverse psychological effects (Watson et al. 1992; Lakeman et al. 2008), proven carriership is likely to be unexpected to couples without a family history. The lessons from Clinical Genetics are that couples should be enabled to consider beforehand what consequences screening might have and whether they are willing and able to accept these, and to anticipate these consequences, especially since couples indicated they would use this knowledge for their reproductive decisions (Lakeman et al. 2008). Here lies an important task for the providers of PCC. In our view, decision counselling regarding preconception genetic screening should address the genetic risks of conceiving an affected child, the possible treatment options, the possibilities to prevent passing on the disease allele, and its consequences, the psychological impact of the various possibilities and the meaning of these possibilities to the couple. Therefore, the PCC counsellor must be skilled in directive and non-directive counselling and must have knowledge of the relevant reproductive options and associated psychological challenges in case of carriership or in case an indication for referral to a Clinical Genetics centre is found.

The PCC counsellor should be aware that genetic and non-genetic risks pose a threat to the idealized pregnancy. A pregnancy, or anticipated pregnancy, fulfils a number of psychological functions (sense of adult identity, enhancement of the self, new object relationship, developmental milestone). Couples may experience tension between the desire to have, nurture and raise a child on the one hand and their sense of responsibility on the other hand. Becoming aware of threats to a desired pregnancy may arouse emotions in the couple, which require attentive counselling. Research is necessary to explore the psychological impact of genetic counselling and offering genetic screening in preconception primary care.
